# ESTABLISHING SHORT-TERM STUDY ABROAD OPPORTUNITIES IN GERONTOLOGY

**DOI:** 10.1093/geroni/igae098.2031

**Published:** 2024-12-31

**Authors:** Linda Johansson

**Affiliations:** Chair

## Abstract

Beginning in 1995, the College of Health and Welfare at Jönköping University has hosted college and university students from the United States in a short-term (10-14 days) study abroad experience in “Healthcare and Social Services across the Lifespan.” Students from multiple U.S. universities have participated, including Pennsylvania State University, Utah State University, and Indiana University Southeast. U.S. students participate with Swedish students in the campus week experiences of an existing course offered by the College of Health and Welfare as part of the primarily online course: “Aging in a Welfare State.” Students from other countries (Finland, Scotland, Spain, Turkey, UK, and Vietnam) have also participated, allowing for comparison of experiences of aging and support for older adults across countries. Symposium presentations will address: Development and content of the study abroad course (Elizabeth Fauth), Facilitators and barriers to establishing a study abroad course at a U.S. university (Carla Hermann), Challenges and benefits of hosting U.S. study abroad students at the College of Health and Welfare (Therése Bielsten), and History of a sustained and successful model of international gerontological education (Steven Zarit). Course outcomes and measures of student satisfaction and success will be presented, as well as feedback from students and faculty and how that shaped the development of the course over time. Plans for future iterations will be addressed. This course model can be replicated by others aiming to promote engaging gerontological education experiences for both U.S. and international students, as well as international research collaborations.

